# It takes two to tango: Ubiquitin and SUMO in the DNA damage response

**DOI:** 10.3389/fgene.2013.00106

**Published:** 2013-06-11

**Authors:** Serena Bologna, Stefano Ferrari

**Affiliations:** Institute of Molecular Cancer Research, University of ZurichZurich, Switzerland

**Keywords:** ubiquitylation, sumoylation, phosphorylation, DNA damage response, cancer therapy

## Abstract

The complexity of living cells is primarily determined by the genetic information encoded in DNA and gets fully disclosed upon translation. A major determinant of complexity is the reversible post-translational modification (PTM) of proteins, which generates variants displaying distinct biological properties such as subcellular localization, enzymatic activity and the ability to assemble in complexes. Decades of work on phosphorylation have unambiguously proven this concept. In recent years, the covalent attachment of Ubiquitin or Small Ubiquitin-like Modifiers (SUMO) to amino acid residues of target proteins has been recognized as another crucial PTM, re-directing protein fate and protein-protein interactions. This review focuses on the role of ubiquitylation and sumoylation in the control of DNA damage response proteins. To lay the ground, we begin with a description of ubiquitylation and sumoylation, providing established examples of DNA damage response elements that are controlled through these PTMs. We then examine in detail the role of PTMs in the cellular response to DNA double-strand breaks illustrating hierarchy, cross-talk, synergism or antagonism between phosphorylation, ubiquitylation and sumoylation. We conclude offering a perspective on Ubiquitin and SUMO pathways as targets in cancer therapy.

## Introduction

The components of signal transduction pathways are organized in a hierarchical manner and communicate with one another. In its simplest formulation, a signaling pathway can be represented with a linear cascade where unidirectional arrows connect a stimulus to the final response through a defined number of intermediates. The recent sequencing of animal and plant genomes and the advent of systems biology have changed this perspective. Proteome scale interaction studies have unveiled the existence of interfaces between pathways and shown that the multiplicity of interactions among their components likely accounts for the array of outputs observed in biological systems. While this novel perspective represented *per sè* a step forward, it still had the intrinsic limitation of merely providing a static snapshot of biological networks. The need for a more realistic picture of signal transduction prompted the development of predictive modeling that, by representing the dynamic flow of information, accounts for the fluctuation of variables as it actually occurs in defined biological systems (Barabasi and Oltvai, 2004). Despite their intrinsic limitation though, “snapshots” provided by reductionist approaches currently represent our best option to study and explain the functioning of signal transduction networks at the molecular level.

Considering that proteins are the constitutive elements of cellular networks and they hierarchically relate to each other, modification of structural or enzymatic traits of one or more elements in a network will necessary affect network properties and result in outputs that are directly observable (i.e., cell proliferation in response to growth factors, cell cycle arrest or terminal differentiation in response to antimitogens or differentiation factors, respectively). Alteration of the properties of network components is achieved through post-translational modifications (PTM), consisting in the covalent addition of chemical groups to one or more amino acids of a protein target in a manner that is, in most cases, reversible. The hierarchical, synergistic or antagonistic combination of PTMs defines a code that translates into distinct outputs.

## Historical perspective

Ubiquitin entered the arena of scientific discoveries in the mid-seventies as result of serendipity and pioneering work initiated in the midst of more trendy studies addressing how the information contained in DNA is decoded to generate the variety of proteins that make up a cell (Ciechanover, 2009). Studies aimed at elucidating the molecular mechanism of liver regeneration led to the identification of a non-histone chromosomal protein, named A24, displaying physicochemical properties similar to those of histones. The localization of A24 in nuclear and nucleolar chromatin as well as its marked decrease upon nucleolar hyperthrophy led to the suggestion that A24 might represent a rDNA repressor (Goldknopf et al., 1975). Ciechanover and colleagues came to the discovery of Ubiquitin from another front. Based on the concept that synthesis and destruction of cellular proteins are homeostatic, with a perfect equilibrium being a necessary condition for life, they undertook studies on mechanisms of protein degradation. Using reticulocytes as model system, that are known to get rid of lysosomes during terminal differentiation but retain the ability of degrading hemoglobin, they set out to identify the non-lysosomal mechanism of protein degradation present in these cells. Using classic biochemical protocols consisting of chromatographic fractionation of crude cell extracts followed by reconstitution of the enzymatic activity of interest through complementation of fractions, they discovered that proteolysis occurs through a cascade of events culminating in the covalent addition of a heat-stable component to proteins targets. Such component was named ATP-dependent proteolysis factor 1 (APF-1) and is now known as Ubiquitin (Ciehanover et al., 1978). Protein modification by APF-1, in turn, was shown to facilitate selective target recognition by the proteolytic machinery (Hershko et al., 1980). The subsequent discovery of several Ubiquitin-like proteins (UBLs) helped shedding light on the complexity of this PTM. UBLs were essentially demonstrated to have functions other than the control of protein degradation. This is the case of the “Small Ubiquitin-like Modifier”, in short SUMO, which was identified as a PTM of RanGAP (Matunis et al., 1996; Mahajan et al., 1997), the activator of the GTPase Ran that controls shuttling of cargos across the nuclear membrane. Sumoylation was shown to facilitate association of RanGAP with the nuclear envelope (Mahajan et al., 1998). Other notable examples are NEDD8, which can be covalently linked to cullins (Hori et al., 1999), the scaffold components of multisubunit Ubiquitin E3-ligases, in a manner that affects their activity; ISG15, which is conjugated to target proteins upon IFNα/β-induced viral response or inflammation (Jeon et al., 2010; Zhao et al., 2013); Urm1, which has low sequence homology to Ubiquitin (Goehring et al., 2003), though it displays a similar fold and is involved in oxidative stress responses in yeast; and, finally, the Atg cascade controlling autophagy in yeast and man, which is the main mechanism responsible for the degradation of cellular components in response to nutrients starvation. This consists of the E1-like enzyme Atg7, the E2-like components Atg3 and Atg7, and the E3-like Atg12-Atg5 conjugate that facilitates transfer of the Ubiquitin-like modifier Atg8 to phospholipids (Hanada et al., 2007).

## Ubiquitylation

**Figure 1:**
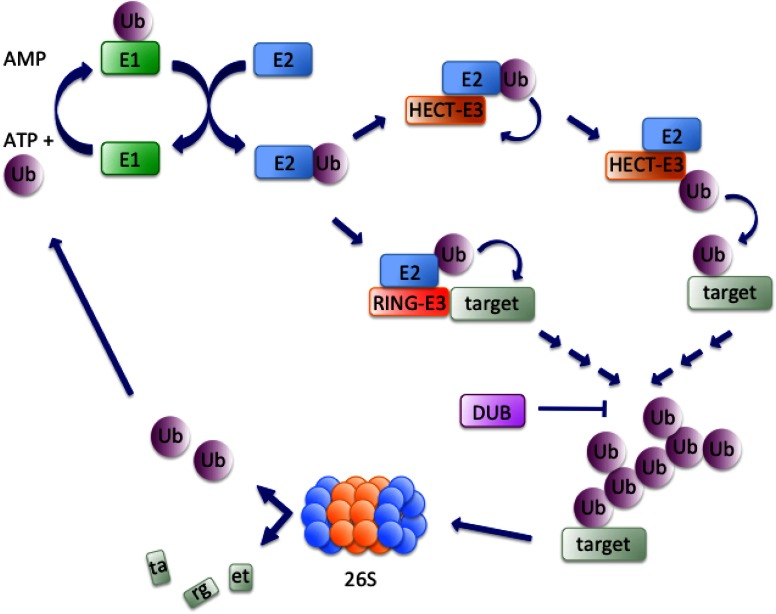
**Simplified scheme of ubiquitylation.** The ubiquitylation cascade initiates with an ATP-dependent reaction consisting in the formation of a thiolester bond between a cysteine in the active site of the E1-activating enzyme and G_76_ in Ubiquitin (Ub). Next, Ubiquitin is transferred to the active cysteine of an E2-conjugating enzyme that interacts with an E3-ligase. The latter can either directly transfer Ubiquitin to lysine residues of the acceptor substrate, as is the case for HECT-E3s, or recruit substrates to the E2 enzyme, a mechanism that characterizes RING-E3s. Finally, ubiquitylated substrates are shuttled to the 26S proteasome and Ubiquitin is recycled for another round of reactions. DUBs oppose substrate degradation by reversing the process of ubiquitylation.

Ubiquitin is a highly conserved regulatory protein of 76 amino acids (8.5 kDa), which is constitutively expressed in all tissues of eukaryotic organisms. In mammalian cells, Ubiquitin is encoded by 4 genes: RSP27A, UBA52, UBB, and UBC (Kimura and Tanaka, 2010). The ATP-dependent conjugation of Ubiquitin C-terminal glycine (G_76_) to lysine residues in the substrate leads to the formation of an isopeptide bond. Ubiquitin itself contains seven lysines behaving as acceptors for additional Ubiquitin molecules to generate poly-chains. Ubiquitylation is carried out in a cascade of reactions: first, a thiolester bond is formed in an ATP-dependent manner between a cysteine in the active site of the E1-activating enzyme and Ubiquitin G_76_. Second, Ubiquitin is transferred to the active cysteine of an E2-conjugating enzyme. Finally, an E3-ligase enzyme binds the E2-Ub complex and transfers Ubiquitin to lysine residues of the acceptor substrate (Hershko and Ciechanover, 1998) (Figure 1). Mammalian cells express only 2 E1s, approximately 38 E2s and more than 600 E3s.

### E2-conjugating enzymes

E2-conjugting enzymes can be classified in 17 subfamilies (Michelle et al., 2009) characterized by an active core called UBC (Ubiquitin-conjugating) domain. Ubiquitin E2 enzymes are structurally similar to UBL modifiers E2s, though the former can specifically interact with the two E1s involved in ubiquitylation (Ye and Rape, 2009). Each E2 enzyme can interact with multiple E3s, as demonstrated for Cdc34 (E2) and SCF complexes (E3) (Skowyra et al., 1997) or the UBE2C/UBE2S (E2s) and the APC/C (anaphase promoting complex/cyclosome; E3) (Williamson et al., 2009) or as shown in network interaction studies (Markson et al., 2009). Specificity is provided by the N-terminal region of the E2 where the amino acidic sequence determines the secondary structure of loops (L1 and L2) that contact two loops and an α-helix of the E3 (Zheng et al., 2000). For E2s interacting with more than one E3, the residue involved in recognition usually differs from one E3 to the other (Zhang et al., 2005a). The binding affinity between Ubiquitin-charged E2s and their cognate E3s is generally high, rendering very fast the kinetic of interaction (Das et al., 2009). Moreover, binding sites for E1 and the specific E3 often overlap in the E2, such that the E2 must dissociate from the E3 to be charged with Ubiquitin by the E1 and vice-versa (Eletr et al., 2005). E2 enzymes catalyze Ubiquitin chains initiation and elongation. Whereas some of them, such as UBE2W and UBE2E in humans, are specifically used by their E3 BRCA1 for chain initiation, the heterodimeric complex UBE2N-UBE2V1 and UBE2K are mainly involved in chain elongation (Christensen et al., 2007; Rodrigo-Brenni and Morgan, 2007; Jin et al., 2008b). Few E2s can mediate both processes, as illustrated by yeast Cdc34 that, together with SCF, is responsible for initiating Ubiquitin chains formation on Sic1 (cell cycle inhibitor subunit of cyclin-dependent kinase 1) in a non-interacting manner and for chain elongation by direct interaction with the substrate (Petroski and Deshaies, 2005).

### E3-ligases

E3s are often part of multimeric complexes and can be divided in two main classes: HECT (Homologous to E6AP COOH-terminus) and RING (Really Interesting New Gene). A cysteine in HECT E3s catalytic domain binds Ubiquitin and transfers it to the substrate in an E2-independent manner (Kulathu and Komander, 2012) (Figure 1). The C-terminal domain of HECT E3s is highly conserved and retains both catalytic activity and the determinants for chain type specificity (You and Pickart, 2001), while the N-terminal region determines substrate specificity (Huang et al., 1999). Established members of the HECT family are E6AP, a partner of the oncogenic E6 protein of human papillomavirus, responsible for p53 downregulation (Huang et al., 1999), Itch/AIP4, with roles in the inflammatory signaling pathways (Chastagner et al., 2006) and Nedd4 and Nedd4L that participate in the development of mouse central nervous system (Kumar et al., 1992).

The vast majority of E3-ligases known to date belongs to the RING family and is characterized by the presence of the Cys/His-rich RING finger domain. The RING finger brings in close proximity substrate and activated E2 enzyme, with the latter directly transferring Ubiquitin to the former (Figure 1). A domain structurally related to the RING finger, the U-box, is found in many E3 ligases of this class (Deshaies and Joazeiro, 2009). Rad18 was the first identified RING domain-containing protein and, together with the E2-conjugating enzyme Rad6, was shown to be essential for post-replicative bypass of UV-induced DNA damage in yeast (Bailly et al., 1997). The RING domain, along with a B-box (zinc-binding fold similar to the RING) and a coiled-coil region (CC), collectively called RBCC supradomain, characterizes the 80 members of the TRIM (Tripartite Interaction Motif) family of E3-ligases (Marin, 2012). A small subfamily of E3 ligases is characterized by the presence of three RING domains: an N-terminal (N-RING), a in-between (IBR) and a C-terminal (RING2 or C-RING) (Eisenhaber et al., 2007). Parkin, a protein involved in Parkinson's disease, is the best-characterized member of this subfamily (Chaugule et al., 2011). The Cullin/RING Ubiquitin ligase (CRL) subfamily represents the largest subgroup of the RING-finger E3 ligases (Deshaies and Joazeiro, 2009). CRLs are multisubunit E3s composed of a RING finger domain protein (Rbx1 or Rbx2) responsible for recruiting the ubiquitylated E2 enzyme, a scaffold protein member of the Cullin family and a receptor for substrate recognition (F-box protein). Some CRLs additionally feature a linker protein, such as Skp1 in the SCF complex or CRL1 and DDB1 in the CRL4 complex (Deshaies and Joazeiro, 2009). CRLs are activated by a PTM consisting in conjugation of NEDD8 to the Cullin component (Pan et al., 2004).

### Atypical ubiquitin chains

Ubiquitylation indicates the process of single Ubiquitin moiety addition to a substrate as well as its extension to form Ubiquitin polymers. Chain extension can occur at all seven lysine residues present on Ubiquitin (K_6_, K_11_, K_27_, K_29_, K_33_, K_48_, K_63_) (Ikeda and Dikic, 2008). E2s such as UBE2N (UBC13) or UBE2RI (CDC34) show specificity for linkage to K_63_ or K_48_, respectively (Vandemark et al., 2001; Petroski and Deshaies, 2005). Others, like UBE2D and UBE2E, can promote different types of Ubiquitin chains formation (Kim et al., 2007). K_48_ and K_63_ linked chains represent the two mostly studied modifications by Ubiquitin, with the first essentially involved in degradation by the 26S proteasome (Komander and Rape, 2012) and the second mainly affecting the function of signaling components (Sun and Chen, 2004) and DNA repair proteins (Chen and Sun, 2009). Proteins undergoing degradation are recognized by the substrate receptor component of the 26S proteasome only if they contain chains longer than four Ubiquitin moieties (Thrower et al., 2000). The processivity of such chains, which is the number of Ubiquitin moieties attached to a protein or to a growing Ubiquitin chain while it is associated to the E3 ligase, determines the rate of substrate degradation (Rape et al., 2006). K_6_-linked chains do not likely have a proteolytic role (Kim et al., 2011). K_11_-linkage, on the contrary, plays a key role in the degradation of cell cycle regulators as well as in endoplasmic reticulum associated degradation (ERAD) and membrane trafficking (Behrends and Harper, 2011). Little is known about the relatively low abundant K_27_-, K_29_- and K_33_-linkages (Komander and Rape, 2012). An additional type of Ubiquitin chain assembly was recently discovered, the so-called LUBAC (Linear Ubiquitin Chains Assembly Complex), which is formed by a complex of two E3 RING-finger ligases, HOIL-1L and HOIP. This type of linkage is characterized by head-to-tail assembly, in which the C-terminal glycine of the previous Ubiquitin is linked to the methionine residue of the next Ubiquitin. Linearly-linked Ubiquitin chains are mainly involved in targeting proteins with a role in innate and adaptive immune signaling pathways (Walczak et al., 2012). Finally, evidence for the presence of more than one linkage type in the same poly-Ubiquitin chain has been provided (Kim et al., 2007). Mixed Ubiquitin chains were shown to result from the activity of the RING finger proteins Ring1B and Bmi1. The latter are components of the Polycomb repressive complex 1 (PRC1), with Ring1B displaying E3-ligase activity toward histone H2A. Mono-ubiquitylation of H2A was shown to depend, at least *in vitro*, from self-ubiquitylation of Ring1B through the generation of atypical mixed K6-, K27-, and K48-based chains on the same Ubiquitin molecule (Ben-Saadon et al., 2006).

### Deubiquitylating enzymes

Ubiquitylation is a reversible process, with deUbiquitinases (DUBs) being responsible for the disassembly of Ubiquitin chains (Nijman et al., 2005). Deubiquitylation controls cell cycle transitions, proteasome- and lysosome-dependent degradation pathways, DNA repair, endocytosis and signal transduction pathways among others. Importantly, DUBs participate in controlling the dynamic state of histone ubiquitylation. An essential function played by DUBs is the co-translational activation of Ubiquitin, which is expressed as fusion to ribosomal proteins or in linear poly-Ubiquitin chains (Reyes-Turcu et al., 2009). A second important function is the recycling of free Ubiquitin from unattached chains (Komander et al., 2009). The human genome encodes approximately 100 DUBs, distinguished in five families: Ubiquitin C-terminal hydrolases (UCH), Ubiquitin specific proteases (USP/UBP), ovarian tumor (OUT), Josephines and JAB1/MPN/Mov34 metalloenzymes (JAMM) (Reyes-Turcu et al., 2009). Whereas the first four families behave as cysteine proteases, JAMM function as zinc-dependent metalloproteases. To prevent inappropriate or unscheduled cleavage of substrates, DUBs activity is controlled by a variety of PTMs, including phosphorylation, ubiquitylation and sumoylation (Reyes-Turcu et al., 2009). Besides the catalytic domain, DUBs feature protein-protein interaction domains and Ubiquitin-binding domains that facilitate formation of multimeric complexes and interaction with substrates, respectively. In most cases, binding to Ubiquitin causes DUBs to undergo conformational changes that expose the catalytic site, which is often hidden by a loop or a larger domain (Reyes-Turcu et al., 2009). DUBs such as USP14, UCH37, and POH1 are often found associated with the 19S subunit of the proteasome, a feature that allows hydrolyzing the poly-Ubiquitin chain from the substrate and recycling Ubiquitin prior to channeling the target protein into the proteasome (Finley, 2009). Reactive oxygen species (ROS) reversibly inactivate Cys-based DUBs, as exemplified by the key regulator of genomic stability USP1, the oxidation of which facilitates PCNA mono-ubiquitylation and the consecutive recruitment of Polη for the repair of oxidation-induced lesions (Cotto-Rios et al., 2012).

### Shuttling to the proteasome

The destiny of proteins modified by K_48_ poly-Ubiquitin chains is degradation by the 26S proteasome. In the DNA damage response, this task is facilitated by shuttling orchestrated by dedicated receptor proteins such as yeast Rad23, Dsk2, Ddi1, and the Shp1/Cdc48/p97 complex. Receptor proteins recognize poly-Ubiquitin chains in their targets by virtue of Ubiquitin-Associated (UBA) domains and interact with subunits of the proteasome via Ubiquitin-Like (UBL) folds, thus effectively shuttling cargoes to the proteasome (Grabbe and Dikic, 2009). The yeast Rad23, which was originally identified for its role in nucleotide excision repair (NER), and its human homologues hHR23A and hHR23B are paradigmatic to this pathway. Rad23 contains two UBA and an N-terminal UBL domain that dynamically interacts with either one of the two UBA domains (Goh et al., 2008). Binding of an UBA domain to poly-Ubiquitin chains of the cargo protein displaces the UBL domain that becomes available for interacting with the proteosomal subunit 5a (Mueller and Feigon, 2003), facilitating the delivery of cargos to the proteasome. Paradigmatic is human p97 and its Ubiquitin-binding partner, the heterodimer UFD1-NPL4, that are recruited to DNA lesions and selectively remove K_48_-Ubiquitin conjugates allowing the subsequent deposition of 53BP1, BRCA1, and Rad51 to regions undergoing repair (Meerang et al., 2011).

## Sumoylation

SUMO proteins and Ubiquitin have only limited sequence identity but they fold in a similar manner (Bayer et al., 1998). SUMO-2 and SUMO-3 are 95% identical but display only 43% identity to SUMO-1. SUMO proteins are generated as inactive precursors and processed by Sentrin/SUMO-specific proteases (SENPs) that catalyze the removal of a C-terminal oligopeptide, exposing the glycine that is conjugated to lysine residues in the target (Xu and Au, 2005).

### Sumo cascade

As for Ubiquitin, SUMO-1, SUMO-2, and SUMO-3 are conjugated to substrates through a dedicated E1-E2-E3 cascade. SUMO proteins bind the activating enzyme E1 [SAE1 and SAE2 in mammals, (Gong et al., 1999)] in an ATP-dependent manner and are transferred to the conjugating enzyme UBC9, which is the only E2 dedicated to SUMO conjugation (Johnson and Blobel, 1997). UBC9 is able to recognize and transfer SUMO to targets in the absence of a co-adjuvating E3, though E3-like proteins containing an SP-RING domain facilitate the process by enhancing the affinity of UBC9 for its substrates (Bernier-Villamor et al., 2002). In the absence of an E3, acetylation apparently provides a means for UBC9 to discern between substrates carrying extended vs. regular recognition motifs (see below) (Hsieh et al., 2013).

The distinct mechanism of SUMO recognition and conjugation likely depends on the different distribution of charged residues on the surface of SUMO proteins as compared to Ubiquitin (Melchior, 2000). Of the SUMO E3-ligases identified to date, some display exquisite specificity, such as RanBP2 that selectively targets RanGAP1 and Sp100 (Pichler et al., 2002). Others, like the PIAS family of proteins that are the mammalian homologues of yeast Siz proteins, act as repressors of STAT3 (Chung et al., 1997) and a number of transcription factors (Schmidt and Muller, 2003). Similarly to RING Ubiquitin ligases, the Siz/PIAS SUMO E3-ligases do not physically bind SUMO but rather interact non-covalently with it. Furthermore, through their zinc-binding SP-RING domain they associate with UBC9. In this manner Siz/PIAS bring SUMO-loaded UBC9 in close proximity to the protein target and facilitate transfer of the SUMO mojety (Hochstrasser, 2001). Among other SP-RING type SUMO E3s, TOPORS was the first reported example of an E3 ligase supporting the transfer of both Ubiquitin and SUMO (Rajendra et al., 2004; Weger et al., 2005).

### Sumo chains

SUMO-2 and SUMO-3 can polymerize to form chains on protein substrates whereas SUMO-1 is only added as monomer (Tatham et al., 2001). It is established that some substrates are modified either by SUMO1, namely RanGAP1 (Saitoh and Hinchey, 2000), or SUMO2/3, namely PML, whereas others are modified indifferently by both SUMO1 and SUMO2/3 (Vertegaal et al., 2006). The reason for such heterogeneity in the SUMO conjugation process is currently unknown, though it may be in part explained by the different pools of SUMO proteins available in the cell, with SUMO1 being mostly conjugated and SUMO2/3 forming a free pool that is mobilized in response to environmental stress (Saitoh and Hinchey, 2000).

The minimal core consensus sequence for recognition and sumoylation of target proteins is defined as Φ-K-X-D/E (with Φ being a hydrophobic residue). An extended sumoylation motif consisting in the sequence Φ-K-X-D/E-X_2_-(E/D)_4−5_ may comprise sites of phosphorylation in the acidic stretch that follows the sumoylated lysine (Yang et al., 2006).

The assembly of proteins complexes in response to sumoylation was addressed by means of two-hybrid screens that led to the discovery of proteins bearing SUMO-interacting motifs (SIMs) (Hannich et al., 2005).

### Desumoylating enzymes

As for other PTMs, sumoylation is a reversible process. The enzymes reversing sumoylation belong to the class of SENP proteins that control SUMO maturation from precursor polypeptides. Of the six SENP enzymes present in the mammalian genome, SENP1 and SENP2 display the ability of their yeast counterpart Ulp1 to control both the maturation of SUMO proteins and desumoylation reactions. SENP1 and SENP2 display a slight preference for pre-SUMO1 or pre-SUMO2/3, respectively, in the process of maturation but act equally well on both during deconjugation (Xu and Au, 2005). SENP3 and SENP5 preferentially remove monomeric SUMO2/3 moieties, whereas SENP6 and SENP7 selectively act on SUMO2/3 chains and do not participate in the maturation of SUMO proteins (Mikolajczyk et al., 2007). SENP enzymes are themselves controlled by sumoylation, ubiquitylation and subcellular localization (Hickey et al., 2012).

### Sumo function

The role of sumoylation at the organism level became apparent thanks to studies in budding yeast showing that depletion of Ubc9 causes cell cycle arrest at G2/M (Seufert et al., 1995). Likewise, studies conducted in fission yeast showed that deletion of the Ubc9 homologue *hus5* is not lethal but results in chromosome segregation defects (Al-Khodairy et al., 1995). Data obtained from chicken DT-40 cells showed that Ubc9 is essential for the viability of higher eukaryotic cells and its knockout results in the formation of multiple nuclei, likely due to cytokinesis defects, with a significant proportion of cells entering apoptosis (Hayashi et al., 2002). Studies conducted in mice confirmed the severe phenotype of Ubc9 knockout, with embryonic lethality observed at early post-implantation stage. Furthermore, blastocysts failed to expand after 2 days in culture and displayed defects in chromosome condensation and segregation as well as dysmorphic nuclear envelopes and disruption of nucleoli and PML bodies (Nacerddine et al., 2005). Sumoylation has also been linked to human pathologies, in that human SUMO1 haploinsufficiency was found to be responsible for cleft lip and palate, a finding corroborated by a mouse model (Alkuraya et al., 2006). Others, however, reported no obvious developmental defects in SUMO1 knockout mice (Evdokimov et al., 2008; Zhang et al., 2008), suggesting possible redundancy among SUMO proteins.

### The sumo enigma

A peculiarity distinguishing SUMO from other PTMs is the ability of triggering fully-fledged responses despite a minor amount of the proteins involved in the response is actually modified by SUMO, a phenomenon denoted as “the SUMO enigma” (Hay, 2005). This occurs in transcriptional repression, where modification by SUMO is apparently required for the recruitment of transcription factors into repressive protein complexes, with their sequestration remaining permanent even upon SUMO removal (Wilkinson and Henley, 2010). SUMO modification of only a small substrate population at any given time point was also suggested to occur for DNA repair proteins such as thymidine-DNA glycosylase (TDG). TDG is part of the base excision repair system (BER) and displays the ability of specifically addressing uracil/thymidine base mismatches (Sancar et al., 2004). The rate-limiting step in the enzymatic reaction carried out by TDG is its dissociation from the abasic site (AP site) generated as first step in the BER process. The high affinity of TDG for the structure generated upon removal of the base is an important self-protection mechanism put in place by the cell since AP sites can turn into DNA strand breaks, thus threatening genome stability (Hardeland et al., 2002). Sumoylation is the appropriate solution to this issue, in that SUMO-modified TDG looses affinity for the abasic site allowing recruitment of the (AP)-endonuclease that acts in the next step of BER (Sancar et al., 2004). To re-initiate the circle, desumoylation by SENPs/ULPs renders TDG promptly available for the next round of lesion recognition and processing (Hardeland et al., 2002). Thus, SUMO modification of minimal amounts of TDG is sufficient to address the repair of uracil/thymidine base mismatches in a highly controlled manner.

## PTMs in DNA damage response: the old and the new

The cascade of events resulting from detection of DNA damage and orchestrating its repair has been best described for DNA double-strand breaks (DSBs). A detailed account of ubiquitylation and sumoylation events occurring at DSBs will be followed by a brief mention to the signaling triggered by other types of DNA lesions.

### DSB recognition

**Figure 2:**
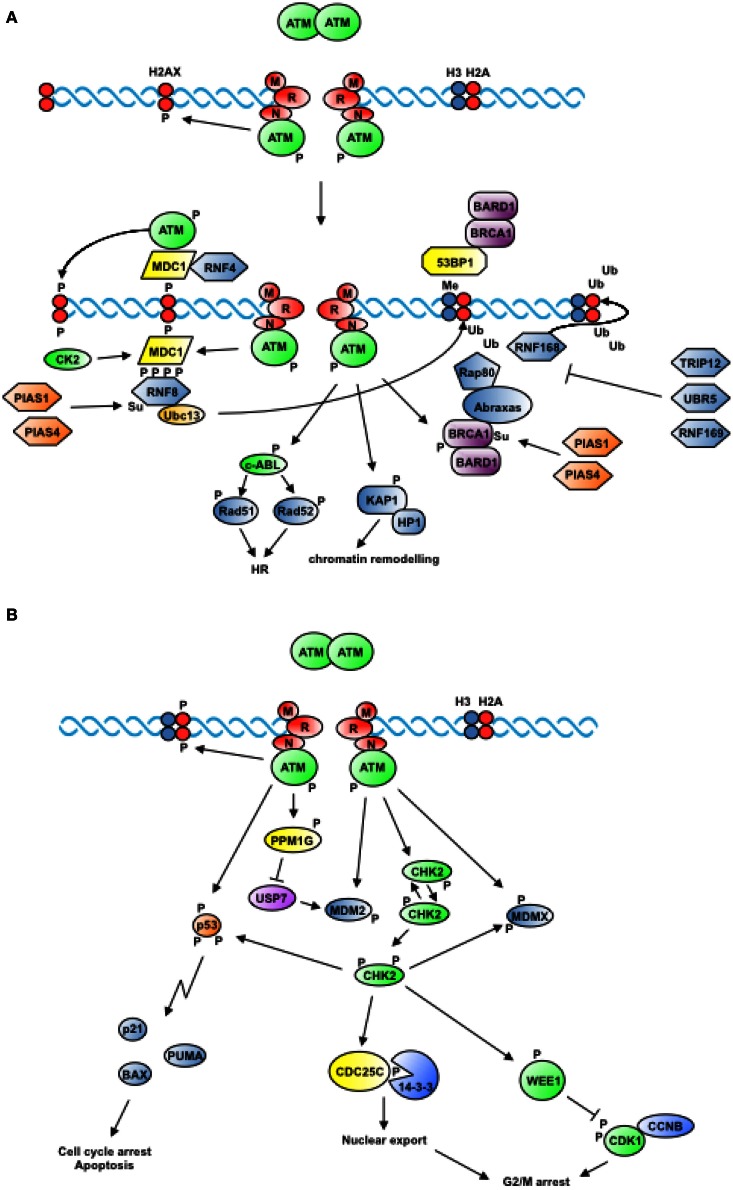
**Proximal and widespread DNA damage signals. (A)** In response to the generation of DSBs, ATM is recruited to DNA in an MRN-dependent manner and is activated by autophosphorylation. ATM-dependent phosphorylation of H2AX triggers the recruitment of factors that mark the site of damage and cooperate to amplify the signal. In addition, ATM phosphorylates proteins that contribute to remodel chromatin and promote homologous recombination (see text for details). **(B)** Activation of ATM triggers the phosphorylation of the protein kinase CHK2 among others, which freely diffuses from the site of damage to transduce DNA damage signals to cell cycle regulators, resulting in the inhibition of cell cycle transitions (see text for details).

Initial players consist of proteins or proteins complexes such as Ku70 and Ku80 or MRE11/RAD50/NBS1 (MRN) that, through recognition and binding to DNA ends, facilitate recruitment and activation of the protein kinases DNA-PKcs or ATM, respectively. The latter function as transducers of the DNA damage signal and help coordinating repair with checkpoint activation and cell cycle arrest (Sancar et al., 2004). When the sister chromatid is available as template, repair is addressed through the error-free pathway of homologous recombination (HR) rather than the predominant but error-prone pathway of non-homologous end-joining (NHEJ) (Sancar et al., 2004). HR initiates upon recognition of DNA ends by the MRN complex, an event that facilitates recruitment of ATM through direct interaction with the C-terminus of the NBS1 component (Falck et al., 2005) (Figure 2A).

ATM is an homodimer and exists in a complex containing the protein phosphatase PP2A, which maintains ATM inactive by catalyzing its constitutive dephosphorylation (Goodarzi et al., 2004), and the histone acetyltransferase Tip60, which is maintained at low level by CUL3-dependent ubiquitylation and plays a role in the modification of chromatin at sites of damage (Murr et al., 2006; Sun et al., 2009). NBS1-dependent ATM recruitment at sites of damage is followed by ATM autophosphorylation at S_1981_ with ensuing activation of the kinase.

The mechanism of DSB repair operating in the absence of a homologous template for recombination-mediated repair is non-homologous end joining. In this case, DNA ends are bound by the Ku70/Ku80 heterodimer that recruits DNA-PK catalytic subunit, causing inward translocation of the heterodimer and positioning the catalytic subunit at DNA ends. Next, depending on the complexity of the lesion, different processing factors are recruited, such as the endonuclease Artemis and the polynucleotide kinase/phosphatase PNKP. The release of DNA-PKcs from DNA ends, which is induced by autophosphorylation, leads to the final step of the process, with XRCC4, DNA ligase IV and XLF performing ligation of the DNA ends (Dobbs et al., 2010).

### Site marking

ATM-mediated phosphorylation of H2AX at the C-terminal S_139_ (γH2AX) (Rogakou et al., 1998), possibly paralleled by dephosphorylation of Y_142_ (Cook et al., 2009), marks the site of damage and contributes to destabilize nucleosome structure (Figure 2A). A critical role in the generation of γH2AX in response to IR is apparently played by mono-ubiquitylation of the histone at K_119_/K_120_, which facilitates the subsequent recruitment of ATM. H2AX mono-ubiquitylation is catalyzed by a complex composed of the polycomb protein BMI1 and the RING finger proteins RING1 and RNF2 (Ginjala et al., 2011; Pan et al., 2011; Wu et al., 2011). Upon phosphorylation, H2AX acts as docking site for MDC1 that, by virtue of the high affinity of its C-terminal BRCT tandem repeats for the phospho-S_139_ epitope in γH2AX, is the first protein localizing at sites of damage (Bekker-Jensen and Mailand, 2010). MDC1 orchestrates the consecutive assembly of factors that will, in turn, mediate the recruitment of DNA repair proteins. Such factors comprise 53BP1, BRCA1, and the E3-Ubiquitin ligase RNF8. Through its N-terminal FHA domain RNF8 binds phosphorylated MDC1 as well as HERC2, with the latter acting as coordinator of Ubiquitin-dependent assembly of DNA repair factors (Bekker-Jensen et al., 2010). SUMO1 modification of HERC2 and RNF168 by the E3-ligase PIAS4 promotes recruitment of RNF8 to the complex and stabilizes the interaction between RNF8 and the E2-conjugating enzyme Ubc13 (Danielsen et al., 2012). In turn, RNF8 contributes to remodel chromatin around sites of damage through a transient K_48_ and a persistent K_63_ ubiquitylation of both H2A and H2AX (Huen et al., 2007; Mailand et al., 2007). Histone ubiquitylation was long known as a post-translation modification occurring during transcriptional responses and mono-ubiquitylation of H2A in the context of the cellular response to DNA damage was first described for the repair of UV-induced lesions (Bergink et al., 2006). RNF8 was identified as the E3-ligase catalyzing H2A and H2B mono-ubiquitylation in response to IR (Wu et al., 2009) and UV (Marteijn et al., 2009) and thus proposed to be a conserved element in the initial response to DNA DSBs and UV lesions. H2A ubiquitylation contributes to the recruitment of DNA repair factors. Once bound to DNA, MDC1 is sumoylated at K_1840_ by PIAS4 in a manner that facilitates its recognition and ubiquitylation by the E3-ligase RNF4, with consequent degradation (Luo et al., 2012). Additional factors recruited to phosphorylated H2AX consist of chromatin remodeling complexes such as INO80 and SWR1 in yeast (Morrison et al., 2004; Van Attikum et al., 2007) and p400 in humans (Xu et al., 2010). K_63_ histone di-ubiquitylation by RNF8, in turn, allows binding of the adaptor protein RAP80 through its UIM motifs (Sato et al., 2009) and the recruitment of Abraxas (ABRA1), which acts as anchor for BRCA1 at sites of DNA damage (Sobhian et al., 2007; Bekker-Jensen and Mailand, 2010). The BRCA1 complex, in turn, contains the DUB BRCC36, which is able to depolymerize K_63_ Ubiquitin chains, thus contributing to maintain steady-state levels of Ubiquitin at sites of damage (Shao et al., 2009).

It has been proposed that initial histone ubiquitylation by RNF8 represents a docking signal for RNF168, a second E3-Ubiquitin ligase that is recruited to chromatin to the purpose of amplifying the signal through further ubiquitylation of histones around the site of damage (Doil et al., 2009; Pinato et al., 2009; Stewart et al., 2009). Structural studies on the RING domains of RNF8 and RNF168 supported this view showing that RNF8 can dimerize and as such productively interact with Ubc13/Mms2 and catalyze K63-linked poly-Ubiquitin chains, whereas the monomeric RNF168 does not interact with the E2 enzyme and is by far catalytically less efficient (Campbell et al., 2012). RNF168 features two MIUs (Motif Interacting with Ubiquitin) that are responsible for recognition of di-ubiquitylated K_63_ on histone H2As and accrual at sites of damage (Doil et al., 2009; Pinato et al., 2009; Stewart et al., 2009). Deletion of the two MIU-domains showed that a small fraction of RNF168 was nonetheless able to bind chromatin (Pinato et al., 2009), leading to the discovery of an additional Ubiquitin-binding domain (UIM- and MIU-related) that is necessary for proper localization of RNF168 at sites of damage (Pinato et al., 2011). A twist to the debate on the hierarchy of E3s recruitment at sites of damage was brought by studies showing that RNF8 is primarily responsible for ubiquitylation of histone H2As at C-terminal sites (K_118_/K_119_), whereas RNF168 catalyzes the mono-ubiquitylation of a set of sites located at the N-terminus of histone H2As (K_13_/K_15_) (Gatti et al., 2012; Mattiroli et al., 2012). This finding indicated that the order by which E3s are recruited does not predict the order in which they ubiquitylate H2As. The authors suggested that RNF168 might catalyze the priming event at N-terminal sites that, being located on the opposite side of the nucleosome with respect to the RNF8 target sites, may initiate distinct signaling events (Mattiroli et al., 2012).

The importance of RNF8-/RNF168-dependent ubiquitylation is well exemplified by the RIDDLE syndrome, where recessive mutations in the *RNF168* gene lead to the expression of aberrant RNF168 protein isoforms, resulting in failure of 53BP1 and BRCA1 accumulation at IR-induced foci and of the subsequent activation of DNA damage responses (Stewart et al., 2009).

Sumoylation of RNF8, RNF168, and BRCA1 mediated by PIAS1 and PIAS4 enhances their E3-ligase activity, contributing to render more efficient histone ubiquitylation at DSBs (Galanty et al., 2009). Through interaction with the Ubiquitin-conjugating UBE2L6/UBCH8, RNF8 controls the degradation of the demethylase JMJD2A/KDM4A resulting in the uncovering of H4K_20_me2 mark and promoting the recruitment of 53BP1 at DNA damage sites (Mallette et al., 2012). RAD18 is another Ubiquitin E3-ligase recruited at DNA lesions through recognition of K_63_ ubiquitylated histones and acting downstream of RNF8/RNF168 (Huang et al., 2009).

The boost of DNA damage-induced ubiquitylation events was initially shown to be modulated by deubiquitylating enzymes such as USP3, BRCC36, and OTUB1 (Nicassio et al., 2007; Shao et al., 2009; Nakada et al., 2010). Subsequent studies on mechanisms that control the excessive spreading of histone ubiquitylation around sites of damage demonstrated the involvement of the HECT-domain E3-ligases TRIP12 and UBR5. By determining the amount of RNF168 that is loaded at sites of damage, TRIP12 and UBR5 contribute to optimize the recruitment of physiological amounts of genome caretakers such as 53BP1 and BRCA1 (Gudjonsson et al., 2012). An RNF168 paralog, namely the E3-ligase RNF169, also contributes to limit the spreading of non-proteolytic ubiquitylation at regions flanking DNA damage sites. Specifically, RNF169 MIU2 domain was demonstrated to recognize histones ubiquitylated by RNF8/RNF168, thus outcompeting and limiting the productive recruitment of 53BP1 and RAP80 (Chen et al., 2012; Poulsen et al., 2012). Recognition of Ubiquitin by RNF168 and RNF169 is mediated by modules composed of a UBD juxtaposed to a short targeting sequence, called the LR-motif (LRM), a structure also shared by RAD18 and RAP80 (Panier et al., 2012).

In concomitance with the events described above, phosphorylation of MDC1 by casein kinase 2 (CK2) allows the former to capture additional molecules of ATM that phosphorylate both H2AX in the neighboring nucleosomes and MDC1 itself (Polo and Jackson, 2011). Phosphorylation does not only serve the function of promoting the assembly of DNA repair modules, but also contributes to break up interactions to facilitate repair processes. This is the case of the transcriptional repressor and RING finger protein KAP1/TIF1β/TRIM28, which is released from chromatin upon ATM-mediated phosphorylation of S_824_, an event that results in the dissociation of heterochromatin protein 1 (HP1) from chromatin and contributes to remodel regions that will undergo repair (Goodarzi et al., 2008). Chromatin relaxation in response to DSBs apparently consists of two stages: an early step that occurs before the generation of γH2AX and that is ATP-dependent (Kruhlak et al., 2006) and a second step that relies on the recruitment of a fraction of the RNF20/RNF40 heterodimer to sites of damage where it catalyzes the mono-ubiquitylation of H2B (Moyal et al., 2011). Mechanistically, it was demonstrated that mono-ubiquitylation of H2B is sufficient to interfere with the compaction of chromatin (Fierz et al., 2011).

### DNA end resection

Marking DNA double-strand break sites is followed by the recruitment of repair proteins in charge of processing DNA ends to create structures that are suitable to recombination. This task is initially accomplished by the MRN complex that in conjunction with CtIP/RBBP8 carries out initial trimming at the break, a step that is followed by extensive processing of DNA ends by the redundant function of EXO1 and the DNA2/BLM complex (Mimitou and Symington, 2009; Eid et al., 2010). Proteins participating in DNA processing are also controlled by PTMs.

CtIP is phosphorylated in a CDK-dependent manner in S and G2 phases of the cell cycle at T_847_ and S_327_. Whereas phosphorylation of the former affects resection activity, modification of the latter influences BRCA1 binding (Yu and Chen, 2004; Huertas and Jackson, 2009). In response to DSBs CtIP is additionally phosphorylated by ATM (Matsuoka et al., 2007). Binding of BRCA1/BARD1 to CtIP is mediated by the BRCT domain of BRCA1 and causes ubiquitylation of CtIP in a manner that does not target it to degradation but facilitates binding to DNA and enrichment at sites of damage (Yu et al., 2006). This is an example of ubiquitylation as means to selectively target protein to a defined region in the cell or to a structure.

In response to stalled replication, EXO1 protein level is controlled by ATR-dependent phosphorylation and poly-ubiquitylation catalyzed by a currently unknown E3 ligase (El-Shemerly et al., 2005, 2008). On the other hand, both in yeast and man EXO1 nuclease activity is controlled by PIKK-dependent phosphorylation upon induction of DSBs (Morin et al., 2008; Bolderson et al., 2010). Sumoylation of EXO1 has also been reported (Tatham et al., 2011), though its functional significance awaits clarification.

The Bloom syndrome helicase (BLM) plays an important role in homologous recombination and in the repair of damaged replication forks (Jones and Petermann, 2012). Modification of BLM by SUMO is necessary for a balanced γH2AX response in HU treated cells, with cells that express SUMO-deficient forms of BLM displaying excessive γH2AX phosphorylation, accumulation of DNA breaks and hypersensitivity to DNA damage (Ouyang et al., 2009). In HU-treated cells expressing SUMO-deficient forms of BLM, the ability to localize RAD51 at damaged replication forks is compromised and sister-chromatid exchanges does not occur. This led to the suggestion that sumoylation represents a switch between pro- and anti-recombinogenic roles for BLM in HR (Ouyang et al., 2009).

Resection of DNA ends by EXO1 or the BLM/DNA2 complex leads to the formation of long 3′-overhangs that are the structures participating in homologous recombination. Replication Protein A (RPA) is the major ssDNA binding protein complex present in eukaryotes and consists of three subunits: RPA1 (70 kDa), RPA2 (32 kDa), and RPA3 (14 kDa). RPA1 has high affinity for DNA and is the docking subunit for a number of proteins involved in DNA synthesis and repair (Fanning et al., 2006). RPA2 has lower affinity for DNA and, thanks to its C-terminal winged helix domain, binds weakly but specifically to AID, to BER proteins such as UDG or to NER proteins such as XPA (Fanning et al., 2006). RPA2 is the major target of phosphorylation events that occur during DNA replication and the DNA damage response. RPA3 is the only component with no affinity for DNA but playing an important role in the stabilization of the trimeric protein complex (Fanning et al., 2006). It has been observed that association between the SUMO protease SENP6 and RPA1 during transition through S-phase maintains RPA1 in a hypo-sumoylated state. Camptothecin-induced DSBs weaken the interaction between RPA1 and SENP6, facilitating RPA1 sumoylation at K_449_ and K_577_, an event that results in increased interaction with Rad51 and displacement of RPA from the ssDNA filament (Dou et al., 2010).

Sumoylation of MRE11 and RAD54 has also been reported (Tatham et al., 2011), though the functional significance of this PTM is as yet unknown.

### Proximal and distal signaling

In addition to the two members of the PIKK family of protein kinases mentioned above in the context of DSB recognition, namely ATM and DNA-PK, also ATR participates in orchestrating the overall response to genotoxic damage. An important component of the DNA damage response is the transduction of signals to the cell cycle machinery. Unlike ATM or DNA-PK that are activated by DNA ends (Uematsu et al., 2007; You et al., 2007), ATR activation specifically depends on the presence of ssDNA resulting from the processing of different types of damage (Zou and Elledge, 2003) or naturally occurring at replication forks (MacDougall et al., 2007). ATR triggering typically occurs after ATM activation (Jazayeri et al., 2006). Two checkpoint kinases are phosphorylated by ATR at S_317_ and S_345_ (CHK1) (Zhao and Piwnica-Worms, 2001) and by ATM or ATR at seven residues in the N-terminal domain (CHK2) (Matsuoka et al., 2000), respectively. This triggers homo-dimerization of the checkpoint kinases and full activation through auto-phosphorylation (Lee and Chung, 2001) (Figure 2B).

The ATR-CHK1 pathway controls the timing of DNA replication origin firing during regular transition through S-phase (Shechter et al., 2004) and triggers G2/M arrest in response to γ-irradiation (Liu et al., 2000). ATR-mediated phosphorylation of CHK1 at S_345_ exposes a degron-like region at the C-terminus of the kinase allowing recognition by cytoplasmic Cul1/FBX6 or nuclear Cul4A/CDT2 SCF E3-ligase complexes that promote poly-ubiquitylation and degradation of CHK1 (Zhang et al., 2009; Huh and Piwnica-Worms, 2013). It has been proposed that proteolysis of activated CHK1 results in checkpoint termination (Zhang et al., 2005b).

The ATM/CHK2 axis controls both transient and sustained cell cycle arrest following detection of DNA damage (Figure 2B) (Shiloh and Ziv, 2013). Namely, by phosphorylating CDC25 phosphatases and the WEE1 kinase, CHK2 blocks cell cycle transitions mediated by Cyclin-CDKs, whereas by phosphorylating p53, MDM2, and PML it promotes apoptosis (Antoni et al., 2007).

Both CHK1 and CHK2 impinge on the machinery driving cell cycle transitions by directly phosphorylating controllers of cyclin-dependent kinases such as the WEE1 kinase and the CDC25A and CDC25C phosphatases (Bartek et al., 2004) (Figure 2B). WEE1 catalyzes phosphorylation of two residues in the Gly-rich P-loop of CDK1, namely T_14_, and Y_15_, in a manner that does not affect nucleotide binding but hampers catalysis (Ferrari, 2006). CDC25 phosphatases specifically remove the phosphate from the two residues in the ATP-binding site of CDKs, causing full activation of Cyclin/CDK complexes (Ferrari, 2006). Inhibition of CDC25C by DNA damage essentially occurs by a 14-3-3-mediated sequestration mechanism, whereas CDC25A degradation via Ubiquitin-proteasome pathways is a primary control mechanism both in dividing cells and in response to DNA damage (Donzelli and Draetta, 2003). Phosphorylation of CDC25A on S_76_ by CHK1 (Jin et al., 2008a) serves as priming event to facilitate phosphorylation on S_79_ and S_82_ by protein kinase CK1 or glycogen synthase kinase-3β (GSK-3β) (Kang et al., 2008; Honaker and Piwnica-Worms, 2010). This, in turn, allows recruitment of the SCF^β − TrcP^ E3 ligase that promotes CDC25A poly-ubiquitylation (Busino et al., 2003).

### DNA damage recovery

Following completion of DNA repair, cell cycle restart is contributed by degradation of molecules that were involved both in signaling DNA damage and in blocking cell cycle progression. This is the case of the adaptor protein Claspin, which is targeted by SCF-β^TrcP^ upon PLK1-dependent phosphorylation (Mamely et al., 2006; Peschiaroli et al., 2006) and whose level is maintained low throughout G1 by the APC/CDH1 E3-ligase (Bassermann et al., 2008), and of the kinase WEE1 (Bartek and Lukas, 2007).

### Other DNA lesions

Additional examples of regulation of DNA damage responses by ubiquitylation are provided by Fanconi Anemia (FA), Translesion DNA Synthesis (TLS) and Nucleotide Excision Repair (NER). FA is an X-linked disease characterized by mutations in genes coding for factors of this DNA repair pathway. Upon exposure to DNA interstrand cross-linking (ICL) agents, FANC proteins form a nuclear “core-complex” in which FANCL is the E3 Ubiquitin ligase responsible, together with its cognate E2 UBE2T, for the mono-ubiquitylation of FANCI and FANCD2 (the ID-complex) on residues K_561_ and K_523_, respectively. This is an event required for the formation of damage-induced foci (Wang, 2007). Mutation of the Ubiquitin-binding domain on FANCI-FANCD2 results in hypersensitivity to mitomycin C or cisplatin (Smogorzewska et al., 2007). In TLS, which represents one of the main mechanisms allowing DNA lesion bypass in S-phase (Waters et al., 2009), ubiquitylation of PCNA plays a key role (see below). BER addresses the repair of modified bases or abasic sites resulting from depurination/depyrimidination events (Almeida and Sobol, 2007). In addition to TDG, which participates in lesion recognition and processing, the BER scaffold component XRCC1 is controlled by phosphorylation (Loizou et al., 2004) and sumoylation (Gocke et al., 2005). The E3-ligase CHIP/STUB1 adds another layer of control to BER by mediating ubiquitylation of the pool of XRCC1 and Polβ that are not directly participating in the process of lesion recognition and repair (Parsons et al., 2008).

## Interdependence of PTMs

### Hierarchical priming

An interesting feature of PTMs is their reciprocal influence, as clearly established for histones, where the antagonism or the synergism of certain modifications defines a “code” that guides protein-DNA interactions (Sims and Reinberg, 2008). These, in turn, influence the compaction of chromatin and ultimately affect biological responses such as transcription, DNA replication and DNA repair (Kouzarides, 2007). Such effects can be cumulative or exclusive, with a clearly defined hierarchy of PTMs affecting a given target protein. In the DNA damage response a notable example of consecutive PTMs occurring in a hierarchical manner is represented by FEN1, the flap endonuclease responsible for cleavage of single stranded 5′ overhangs in Okazaki fragments during DNA replication and also involved in DNA repair. Phosphorylation at S_187_ in FEN1 catalytic domain by cyclin A/CDK2 results in its release from PCNA, the DNA polymerase processivity factor that stimulates FEN1 nuclease activity (Henneke et al., 2003). Subsequent modification of K_168_ in FEN1 by SUMO3 facilitates K_354_ ubiquitylation by the E3 ligase PRP19, resulting in FEN1 degradation at the end of S-phase, an event that contributes to ensure a timely transition to G2 (Guo et al., 2012).

### Competition for the substrate

In addition to the ability of sumoylation to directly alter the properties of the protein undergoing this modification, it may also serve as a competitor to other PTMs. Indeed, since sumoylation targets lysine residues in the substrate, similarly to ubiquitylation, methylation or acetylation, the modification of one or more lysine in the substrate could block other PTM machineries from accessing these residues, thus indirectly affecting protein function (Walsh et al., 2005). Established examples of competition among PTMs are RanGAP1, which upon sumoylation preferentially binds the nuclear pore complex (Melchior, 2000), the NF-κB signaling pathway (Huang et al., 2003) and PCNA (Hoege et al., 2002). Specifically to the latter, early studies showed that mono-ubiquitylation mediated by RAD6 (E2) and RAD18 (E3), K_63_ poly-ubiquitylation by MMS2, UBC13, and RAD5 and sumoylation by UBC9 all affect the same lysine residue (K_164_) (Hoege et al., 2002). Subsequent work clearly established that PCNA mono-ubiquitylation supports translesion synthesis, a pathway allowing stalled DNA replication to proceed beyond damage through the replacement of processive polymerases with specialized polymerases (Bienko et al., 2005; Garg and Burgers, 2005). K_63_ poly-ubiquitylation, on the other hand, facilitates synthesis by a template-switch mechanism, a complex but essentially error-free pathway that utilizes the undamaged, newly synthesized daughter strand of the sister chromosome as template (Branzei and Foiani, 2010). Finally, PCNA sumoylation prevents the formation of DSBs and the occurrence of inappropriate recombination events at stalled DNA replication forks by a mechanism involving the anti-ricombinogenic activity of the helicase Srs2 in yeast (Papouli et al., 2005; Pfander et al., 2005) and possibly by a similar mechanism in humans (Gali et al., 2012).

### Cross-talking

A number of Ubiquitin E3-ligases display the ability to bind SUMO chains on proteins that, in turn, become their substrates. A reported case is PML, which undergoes modification by SUMO-1 as well as by SUMO-2/3. Whereas attachment of SUMO-1 determines confinement of the protein in PML nuclear bodies (Muller et al., 1998) formation of SUMO2/3 chains facilitates the recruitment of the E3-ligase RNF4, which ubiquitylates the SUMO chains and ultimately targets PML to degradation (Lallemand-Breitenbach et al., 2008; Tatham et al., 2008; Weisshaar et al., 2008). RNF4 displays the ability to interact with other sumoylated substrates, such as MDC1 and RPA, via its N-terminal SUMO interaction motif (SIM) and to subsequently regulate their stability (Galanty et al., 2012). Another interesting case is BRCA1, which co-localizes with and is sumoylated by PIAS1 and PIAS4 at sites of damage. This, in turn, was reported to enhance BRCA1 E3-ligase activity possibly through a SUMO-dependent increase of the E3-E2 interface (Morris et al., 2009).

### Synergy

The advent of proteome-wide studies allowed appreciating the fact that, like phosphorylation, sumoylation triggered by a defined stimulus or stress does not target a single components but a vast majority of the protein machinery involved in the response. Work conducted in yeast established that lack of overall sumoylation in a hypomorphic mutant of the SUMO E2 Ubc9 impaired survival in response to DNA damage (Cremona et al., 2012). This resulted from incomplete replication of damaged DNA as well as defective resection at DSBs. The authors found that DNA damage-induced sumoylation occurred independently of phosphorylation events that were triggered by the checkpoint and was proposed to act in parallel with them to support cell survival (Cremona et al., 2012). A study conducted using SILAC-based mass spectrometry identified 844 different SUMO conjugates, the abundance of which did not seem to change in response to DNA damage (Psakhye and Jentsch, 2012). Interestingly though, the set of sumoylated proteins enriched in response to DNA damage was specifically that of the HR machinery. The authors found that DNA end resection and the consecutive generation of long ssDNA tracts acted as trigger to the wave of sumoylation that characterized the response. Sumoylation of HR proteins was found to occur independently and in parallel, with no influence of one sumoylation event on the other, and to entirely depend on the SUMO E3-ligase Siz2. Sumoylation promoted physical interaction among HR proteins, thus facilitating DNA repair (Psakhye and Jentsch, 2012).

## Ubiquitin and sumo as targets in cancer therapy

Ubiquitylation and/or sumoylation defects have been implicated in the pathogenesis of a number of human diseases among which is cancer (Sun, 2006; Bettermann et al., 2012).

### Ubiquitin and cancer

Examples of over-expression of ubiquitylation pathway components in cancer cells are the p53-specific ARF-BP1/Mule HECT E3-ligase, the F-box proteins SKP2 and β-TrcP1, the SCF component Cul-4A and the RING-finger proteins RNF11, ZNF164 (Chen et al., 2006), and RNF5 (Bromberg et al., 2007). Mutation or deletion of E3-ligases that normally function as tumor suppressors has also been reported. This is the case of the RING-finger E3-ligases BRCA1/BARD1 and SIAH1 (Chen et al., 2006). Finally, epigenetic inactivation of genes coding for the E3-ligase HACE1 (Hibi et al., 2008) or the RING-finger protein CHFR (Chen et al., 2006) has been observed in several types of carcinomas.

Based on the reasoning that E3 enzymes are druggable targets, pharmaceutical companies embarked on high-throughput screenings in search for compounds that would target the active site of E3-ligases or block interaction with their substrates (Sun, 2006; Hoeller and Dikic, 2009). A notable example of the latter is Nutlin, which impairs the p53-HDM2 interaction by filling a groove in HDM2 where p53 is accommodated (Vassilev, 2007). Despite the initial enthusiasm raised by Nutlin and its derivatives, the limitation of its efficacy in cells expressing wild-type p53 excluded their use from a number of other cancers. More discouraging, the cytostatic effect of Nutlins in p53-deficient cells indicated that they did not solely inhibit the p53/HDM2 interaction (Vanderborght et al., 2006). The p53-targeting molecule RITA (NSC652287), identified in a screening conducted on a pair of isogenic cell lines differing only in their p53 status, was shown to bind p53 N-terminus (Issaeva et al., 2004). However, RITA did not specifically target the p53-HDM2 dimer but also other p53 protein complexes (Hjerpe and Rodriguez, 2008). Similar issues were encountered with other inhibitors of E3-ligases (Guedat and Colland, 2007).

Another interesting case of targeting E3-ligases is BRCA1. Synthetic lethality was observed when PARP-inhibitors are administered to cells of BRCA-deficient patients (Bryant et al., 2005; Farmer et al., 2005). Considering that BRCA deficiency occurs in <5% of breast cancers, the results obtained with PARP-inhibitors prompted studies attempting to exploit the concept of synthetic lethality in non-mutation carriers. Specifically, small molecules targeting the phospho-dependent interaction of BRCA1 with partners such as Abraxas were administered to breast and cervical cancer cells to mimic the inactivating mutation of the otherwise wild-type BRCA1 gene. The data showed that, under these conditions, PARP-inhibitors effectively sensitized cells to IR-induced damage (Pessetto et al., 2012).

The E1-activating enzyme and the proteasome have also been considered as possible targets, with the *caveat* that inhibiting the ubiquitylation cascade at its apex may impair pathways of vital importance to the survival of normal cells. This is particularly true if one considers the widespread use of ubiquitylation in the control of cellular functions. Nonetheless, inhibitors of the chymotryptic activity of the proteasome have been identified and characterized. Compounds such as bortezomib have received approval from FDA and are currently used for the treatment of multiple myeloma and mantle cell lymphoma (Guedat and Colland, 2007; Rastogi and Mishra, 2012). Similarly, ATP-competitive inhibitors blocking the transfer of Ubiquitin from the E1-activating enzyme to E2-conjugating components of the cascade have been identified (Guedat and Colland, 2007).

Inhibition of deubiquitylating enzymes has also been explored as possible alternative to the development of inhibitors of the ubiquitylation cascade. A compound specifically targeting USP7 was shown to stabilize p53, activate p53-dependent transcription, block cell growth and induce apopotosis (Guedat and Colland, 2007). Recently, a novel strategy based on the use of combinatorial libraries of Ubiquitin variants has led to the identification of mechanisms of DUBs inhibition and provided the demonstration that this approach could be applied to the discovery of specific E2 or E3 inhibitors (Ernst et al., 2013).

### Sumo and cancer

With regard to the role of SUMO in cancer, Ubc9/UBE2I was found overexpressed in ovarian carcinoma specimens (Mo et al., 2005). Xenografts studies conducted in mice revealed that tumors expressing wildtype Ubc9 grew better than controls, while tumors expressing dominant negative Ubc9 exhibited reduced growth (Mo et al., 2005). A comprehensive study reported an increase in UBC9 expression in primary colon and prostate cancer compared with their normal tissue counterparts, whereas UBC9 levels were found lower in metastatic breast, prostate, and lung cancer in comparison with their corresponding normal and primary adenocarcinoma tissues (Moschos et al., 2010). Increased UBC9 expression was also observed in melanoma-infiltrated lymph nodes, with depletion of UBC9 resulting in sensitization of melanomas to the cytotoxic effects of topotecan and cisplatin (Moschos et al., 2007). A comprehensive collection of studies on UBC9 mRNA expression pattern in different cancer types can be found at www.nextbio.com. Based on these findings, targeting UBC9 in cancer therapy was initially proposed (Mo et al., 2005). However, given the widespread use of sumoylation as PTM controlling numerous metabolic pathways, altering the overall pattern of sumoylation in the cell was countered by others as a non-specific and ineffective method to combat cancer (Bawa-Khalfe and Yeh, 2010). Support to arguments in favor of UBC9 as valid target in cancer therapy is provided by its pattern of differential expression, with higher levels of UBC9 in cancerous vs. normal tissues, offering a possible therapeutic window (Mo and Moschos, 2005). In this respect, crystallographic studies mapping the surfaces in UBC9 involved in the interaction with specific E3s and their substrates represent a promising avenue to the design of small compounds disrupting selective sumoylation reactions (Mo and Moschos, 2005).

Increased levels of the desumoylating enzyme SENP1 were reported in thyroid oncocytic adenocarcinoma (Jacques et al., 2005) and prostate cancer (Cheng et al., 2006). A transgenic mice model showed that overexpression of Senp1 in the prostate led to the development of prostatic intraepithelial neoplasia at an early age (Cheng et al., 2006). Promising results have been obtained in studies aiming at the identification of SUMO-specific protease (SENP) inhibitors (Hemelaar et al., 2004; Borodovsky et al., 2005) or based on the screening of cysteine-protease inhibitor libraries (Albrow et al., 2011). The latter, in particular, led to the identification of two classes of compounds: the first, containing a reactive aza-epoxide electrophile linked to an extended peptide backbone and the second, containing an acyloxymethyl ketone reactive group. Structure-activity relationship studies led to the design of covalent inhibitors of multiple hSENPs displaying micromolar IC_50_ values (Albrow et al., 2011).

Arsenic trioxide, which induces differentiation of leukemic blasts and clinical remission, was shown to promote SUMO-dependent poly-ubiquitylation of PML-RARα by the Ubiquitin E3-ligase RNF4, with consequent degradation of the fusion protein responsible for acute promyelocytic leukemia (Lallemand-Breitenbach et al., 2008; Tatham et al., 2008). Thus, in addition to classic approaches based on the chemical inhibition of enzymatic activity, the case of arsenic trioxide illustrated that among the variety of possible avenues to inhibit function, the exploitation of existing pathways in the cell that may be triggered at will is an important option.

### Conflict of interest statement

The authors declare that the research was conducted in the absence of any commercial or financial relationships that could be construed as a potential conflict of interest.
